# Reactivity and Microstructure of Metakaolin Based Geopolymers: Effect of Fly Ash and Liquid/Solid Contents

**DOI:** 10.3390/ma12213485

**Published:** 2019-10-24

**Authors:** Oliver Vogt, Neven Ukrainczyk, Conrad Ballschmiede, Eddie Koenders

**Affiliations:** Institute of Construction and Building Materials, Technische Universität Darmstadt, 64287 Darmstadt, Germany; ukrainczyk@wib.tu-darmstadt.de (N.U.); ballschmiede@wib.tu-darmstadt.de (C.B.); koenders@wib.tu-darmstadt.de (E.K.)

**Keywords:** geopolymer, metakaolin, fly ash, reactivity, microstructure, Si/Al ratio, MIP, SEM-EDS, TGA-DSC

## Abstract

Geopolymers are inorganic binders based on mixtures of an aluminosilicate powder with an alkali-silicate solution. Properties of geopolymers are strongly determined by the type of reactive solid, the liquid/solid ratio of paste and, amongst others, the Si/Al ratio of the formed geopolymer network. In this study, fly ash blended metakaolin based geopolymers with varying liquid/solid ratios (l/s), activated by potassium silicate solution, are investigated. Reactivity of metakaolin and fly ash was investigated by powder X-ray diffraction (XRD) and dissolution tests. Reactivity, mechanical properties and microstructure of hardened pastes were analyzed by setting and compressive strength tests, mercury intrusion porosimetry (MIP), capillary water absorption tests, thermogravimetric analysis-differential scanning calorimeter (TGA-DSC), isothermal calorimetry and scanning electron microscopy with energy dispersive spectroscopy (SEM-EDS). The results show that substitution of metakaolin by fly ash as well as variation of l/s brings advantages up to a certain degree, but also has a considerable influence on the pore size distribution, mechanical properties, Si/Al ratio of the geopolymer network and the content of bound water.

## 1. Introduction

For the synthesis of geopolymers with low to no calcium content, metakaolin is the most used powdery solid material if hardening has to take place at room temperature. The low calcium content is the decisive feature that distinguishes geopolymers from alkali activated binders (AAB) in general, a fact that is based on the definition for geopolymers coined by Joseph Davidovits in 1979 [1], after activating metakaolin with alkali silicate solution [2]. 

Literature mentions typical material properties for geopolymers, such as high chemical resistance, rapid hardening with high final strengths, high temperature resistance and the ecological advantages of the inorganic binder. However, these general statements do not apply to all geopolymers, as a large number of raw materials are available for the synthesis of this type of binder and their composition and reactivity have a major influence on the properties of the final product. [3] In this context, the Si/Al ratio of the raw material composition must be mentioned as one of the most decisive criteria which influences, among other things, the solidification [4], the strength [5,6] and the acid resistance [7] of the geopolymer. The increased acid resistance compared to Portland cement based binders is one of the most frequently mentioned advantages of geopolymers [7,8,9,10,11,12].

When the powdery solid comes into contact with the alkaline solution, hydroxide (OH^−^) of the alkaline solution breaks the bonds of the silicon and aluminum species of the powdery precursor and in the course of the subsequent polycondensation reaction, the dissolved species form the geopolymer network [13]. Such network consists of aluminate and silicate tetrahedrons, cross-linked via “oxo” (-O^2-^-) bridging bonds [14]. The alkali cations (Na^+^, K^+^) of the alkaline solution are integrated to a certain degree into the network to balance the excess negative charge of the aluminate tetrahedrons [4]. Due to the favorable ratio of silicon to aluminum (Si/Al) in metakaolin it is an ideal solid for the synthesis of geopolymers [15,16], as the Si/Al ratio plays an equally decisive role in addition to the total content of reactive phase [17]. The high reactivity of the metakaolin precursor also results from the morphology and high specific surface area of the calcined particles [18], as well as the position and high content of hydroxyl groups in the structure of Kaolinite, the precursor of metakaolin [19]. The morphology of metakaolin, however, results in a high water demand of the powder [18] and a high viscosity of the geopolymer paste [20]. Nonetheless, geopolymers based on metakaolin can have a high durability with regard to carbonation and also alkali-silica reaction, which has been demonstrated by Pouhet [21]. For fly ash geopolymers, in most cases a temperature post treatment becomes necessary [22,23,24,25,26,27,28,29,30,31,32,33]. An important technological challenge for the application of fly ash geopolymers is the high variability in quality of the ash itself [34,35,36]. Furthermore, the microstructure of fly ash based geopolymers differs largely from aluminosilicate networks based on metakaolin [37]. The decisive advantage of fly ash geopolymers is the significantly lower water demand of the powder and the resulting lower porosity of the hardened geopolymer [29]. Within a hardened geopolymer network the bound water results, among other things, from the charge-balancing alkalis since the cations Na^+^ and K^+^ are bound into the network in hydrated form [38]. Since most of the liquid phase from the alkaline solution is not bound inside the network this results in relatively higher porosity compared to conventional cement based materials. By using fly ash and lower liquid/solid ratios (l/s) ratios, a denser geopolymer can be achieved. In order to obtain approximately the same workability of fresh geopolymer pastes Kong et al. [39] applied a l/s ratio of 0.33 for fly ash based geopolymers whereas 1.25 for metakaolin based ones almost doubled the porosity of the geopolymer. To tackle the challenge of the high water demand for metakaolin based geopolymers, additional fly ash can be used to improve rheology and also optimize the strength development [18,40,41]. By applying mixtures of both metakaolin and fly ash, metakaolin acts as a source for the required content of reactive phases, whereas fly ash causes an increase in workability or reduces the total water demand of the powder mixture, which reduces also porosity and increases the density of the hardened geopolymer [42]. Replacing metakaolin by fly ash has been investigated in some publications [41,43,44,45], where mostly metakaolin with a very high fineness was used, which resulted in very high initial l/s ratios. The low l/s ratio of 0.4, as applied by Duan et al. [43], could only be realized at very high fly ash contents (> 80%). If the fly ash content is varied parallel to the l/s ratio, as done by Zhang et al. [44], properties of hardened geopolymer were shown to change when adapting the workability. However, the direct influence of substitution by fly ash was difficult to interpret. Zhang et al. [45] applied a wide range of l/s ratios, nevertheless, the maximum amount of fly ash in the powder mixture was only 20%. Substitution of metakaolin by fly ash up to 50% at constant l/s ratio was investigated by Zhang et al. [41], with an optimal substitution rate of 10% being mentioned.

The aim of this work is to quantify both the influence of fly ash and the effect of the l/s ratio on the properties of fresh but mostly hardened metakaolin based geopolymers. The used metakaolin is an impure one with relatively high proportion of quartz. By using three different l/s ratios as well as six substitution rates of fly ash effects in workability, setting, porosity, strength evolution and composition of the geopolymer microstructure were investigated in the following sections. 

## 2. Materials and Methods 

### 2.1. Materials

As powder precursors, an industrial (quartz-rich) metakaolin and a standard fly ash (Class F) were used. Metakaolin consists mainly of SiO_2_ (68.0%) and Al_2_O_3_ (27.0%), with a minor content of Fe_2_O_3_ (2.4%) and CaO (0.8%). Fly ash contains 52.6% SiO_2_, 25.1% Al_2_O_3_, 8.0% Fe_2_O_3_, 3.0% CaO and some other components to a significantly lesser extent. The specific surface area of metakaolin is 0.99 m^2^/g with a medium grain size of 41.4 µm and 0.81 m^2^/g for fly ash with a medium grain size of 38.5 µm, respectively. As alkaline activator an industrial potassium silicate solution with a molar SiO_2_/K_2_O ratio of 1.5, a solid content of 45%, a viscosity of 20 mPas, a density of 1.51 g/cm^3^ and a pH of 13.5 was used.

### 2.2. Characterization Methods for Powder Precursors

Crystalline phases of metakaolin and fly ash were determined by quantitative X-ray powder diffraction (Bruker D2 Phaser, Hamburg, Germany), using Rietveld refinement in DIFFRAC.TOPAS software (Version 5, Bruker), which is an approved method for quantifying crystalline phases [44]. Corundum (10% spiked samples) was used as an internal standard. Rietveld quantification of amorphous phases was done by considering the broad humps in powder X-ray diffraction (XRD) spectra, a method that has been applied in literature before [45,46]. Characteristic humps indicating amorphous phases for metakaolin are with 2θ between 18° and 38° [47], respectively between 20° and 30° for fly ash [48]. 

Reactivity of metakaolin and fly ash was measured by performing dissolution tests, where 150 mg of powder was immersed in 150 g of potassium hydroxide solution and continuously stirred with a magnetic stirrer. To receive information about the influence of the testing procedure, the concentration of potassium hydroxide solution (10%, 20%), the temperature (60 °C, 21 °C) and the duration (24 h, 6 h) was varied. Four different types of dissolution tests with different boundary conditions (BC1, BC2, BC3 and BC4) were performed. Eluates were filtered and analysed by inductively coupled plasma optical emission spectroscopy (ICP-OES) using an Optima 2000 DV (Perkin Elmer, Waltham, MA, USA). Although the geopolymers were activated with potassium silicate solution, potassium hydroxide solution was used for the dissolution tests. This allows the actually dissolved Si and Al species from the powder to be determined, without dissolved Si of the potassium silicate solution affecting the results or possible oligomer formations falsifying the Si and Al proportions quantifiable by means of ICP-OES. The same approach was adopted by Buchwald [49], among others. Compared to potassium hydroxide solution, potassium silicate solutions does not accelerate the dissolution process, but accelerates solidification and hardening, since Si of the potassium silicate solution is involved in the polycondensation reaction [50] by forming a first alumosilicate gel with the Al species dissolved from the powdery precursors [51]. Since the polycondensation reaction is not the aim of the dissolution tests, potassium silicate solution is not required.

Particle size distribution and specific surface area of metakaolin and fly ash was measured by static laser light scattering in ethanol, using a particle size distribution analyzer (LA-950V2, Retsch, Haan, Germany).

### 2.3. Manufacture of Geopolymers

Within the scope of this study, 18 different geopolymer paste types were investigated to analyze the influence of the substitution of fly ash for metakaolin (0, 10, 20, 30, 40 and 50 wt.%) as well as various l/s ratios (0.49, 0.54 and 0.60), where “l” comprises the total weight of potassium silicate solution and “s” the weight of metakaolin and fly ash. The labeling of the mixtures contains both the mass content of fly ash in the powder mixture and the l/s ratio. For example, “FA10.49” has a mass fraction of fly ash of 10% (accordingly 90% of metakaolin) and an l/s ratio of 0.49. “MK.49” contains only metakaolin with an l/s ratio of 0.49. The geopolymers were produced by dry mixing metakaolin and fly ash in a first step before adding potassium silicate solution, and subsequently mixing for 10 minutes with a standard planetary mortar mixer (E092-01N, Mixmatic). Pastes where cast in prism molds (160 mm × 40 mm × 40 mm) and vibrated until no more air bubbles could be seen on the surface. Specimens were demolded after 1 day and wrapped in aluminum adhesive tape in order to avoid moisture loss and stored at 21 °C and a relative humidity of 50% up to the date of characterization. 

### 2.4. Characterization Methods for Fresh Geopolymers

Workability of fresh paste was determined according to DIN EN 1015-3 by spread-flow test, but without operating the spindle of the table. The air void content of the fresh pastes was measured with an Air Entrainment Meter (1 dm^3^ volume, FORM+TEST) according to DIN EN 12350-7. Isothermal calorimetry was conducted at 21 °C with a cement calorimeter (MC CAL, C3 Prozess- und Analysentechnik, Haar, Germany) using an in-situ mixing device. The setting time of the pastes was determined by using an automatic Vicat needle instrument (ToniSET One, Toni Technik, Berlin, Germany). 

### 2.5. Characterization Methods for Hardened Geopolymers

All characterization methods for hardened geopolymers were performed on 28 day cured samples. Compressive strength, additionally determined after 1 day, 7 days and 56 days of curing, was tested with half prisms (80 mm × 40 mm × 40 mm) according to DIN EN 196-1 at a load increase of 2.4 kN/s. Skeleton density of the geopolymers, as well as bulk density of metakaolin and fly ash was measured with a Pyknomatik-ATC (ThermoFisher, Waltham, MA, USA). Mercury intrusion porosimetry (MIP) measurements were conducted with a Pascal 440 Mercury Porosimeter (ThermoFisher). To stop the reaction and remove the water from the specimens before MIP measurements, samples were immersed in liquid nitrogen and kept in a freeze dryer (Lyotrap, LTE Scientific Ltd, Oldham, UK) until mass constancy was achieved. Water adsorption test for determining the free capillary porosity of specimens was performed on dried cubic samples (4 cm × 4 cm × 4 cm). To measure the mass loss of hardened geopolymer powder samples up to 1000 °C, TGA-DSC (thermogravimetric analysis-differential scanning calorimeter) was performed with a STA 449 F5 Jupiter (Netzsch, Selb, Germany). Therefore, geopolymers were crushed and grinded with acetone which led to rapid evaporation of the water in the sample and to a stop of reaction, a method comparable to storing test specimens in acetone [46] or ethanol/acetone mixtures [47] to stop the reaction. For each measurement 35–40 grams of dry powder was heated up to 1000 °C at a heating rate of 20 °C/min. Scanning electron microscopy with energy dispersive spectroscopy (SEM-EDS) was performed with a Zeiss EVO LS25 SEM (Jena, Germany) and an EDS detector (EDAX, Ametek, Berwyn, PA, USA) with an accelerating voltage of 15 keV and a beam current of 2.0 nA. SEM-EDS was used as a semi-quantitative technique to analyze the Si/Al ratio of the alumosilicate network of the geopolymers. To determine the mean value and the standard deviation of the Si/Al ratio, 20 spots of geopolymer gel were analyzed for each specimen.

## 3. Results

### 3.1. Reactivity of Metakaolin and Fly Ash

Total amorphous amount and crystalline phases are shown in Table 1. Metakaolin contains 46.0% amorphous phases, additional crystalline phases are mainly quartz, muscovite and mullite. The amorphous content of fly ash (72.0%) is significantly higher than that of metakaolin, crystalline phases are quartz, mullite, hematite and magnetite. Further crystalline phases to a lesser extent are sanidine (NA), tosudite and illite for metakaolin resp. hematite and magnetite for fly ash.

By taking into account the chemical composition of metakaolin and fly ash and the crystalline and amorphous phases, the total amount of amorphous Si and Al for both materials was calculated, a method which is used in literature to calculate the amorphous composition of fly ash [48,49,50] and metakaolin [21]. The amorphous content of Si results from the difference of the total content of Si in the solid, from chemical composition of powder precursors, and the crystalline content of Si in silicate minerals like quartz from powder X-ray diffraction (XRD) analysis. Considering the molar masses of Si and Al, a molar ratio Si/Al was calculated. Table 2 shows the amorphous amounts of Si and Al, the calculated molar Si/Al ratio, as well as the amount of amorphous SiO_2_ and Al_2_O_3_. It must be mentioned in this context that the accuracy of the results strongly depends on the testing procedure (e.g., internal standard for spiked samples and setting of XRD device) and evaluation of the results (e.g., different mineral compositions of quartz and mullite) [50]. Therefore certain minor inaccuracies in the results cannot be excluded. However, the results prove the clear difference in the amorphous composition of the two powdery precursors.

**Figure 1:**
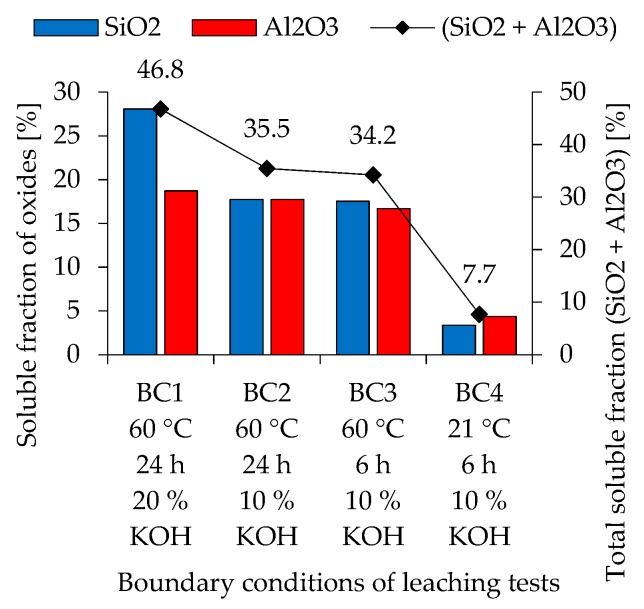
Soluble fraction of oxides and total soluble fraction of oxides of metakaolin, obtained from ICP-OES (inductively coupled plasma optical emission spectroscopy) analyzes of eluates.

**Figure 2:**
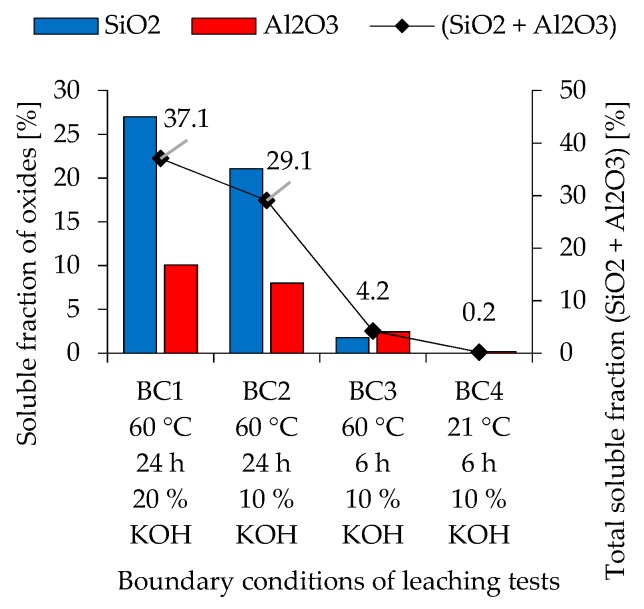
Soluble fraction of oxides and total soluble fraction of oxides of fly ash (ICP-OES of eluates).

Soluble fractions of SiO_2_, Al_2_O_3_ and the total soluble fraction (SiO_2_ + Al_2_O_3_) of metakaolin are shown in Figure 1, and for fly ash in Figure 2, as was calculated from the dissolution test results. Metakaolin exhibits higher reactivity than fly ash. For both precursors, higher concentrated potassium hydroxide solution, elevated temperature and longer stirring time leads to higher amounts of dissolved species. 

For metakaolin, a significantly higher amount of SiO_2_ gets dissolved, when a higher concentrated potassium hydroxide solution is used (BC1: 60 °C; 24 h; 20% KOH), compared to the dissolved Al_2_O_3_. For BC2 (60 °C; 24 h; 10% KOH) and BC3 (60 °C; 6 h; 10% KOH), the total soluble fraction decreases from 46.8% (BC1) to 35.5% (BC2) resp. 34.2% (BC3). In contrast to BC1, however, BC2 and BC3 have approximately the same amount of dissolved SiO_2_ and Al_2_O_3_. Changing the temperature from 60 °C to 21 °C (BC4: 21 °C; 6 h; 10% KOH), the total soluble fraction decreases significantly. The dissolution of Al_2_O_3_ is, apart from BC4, almost constant under all boundary conditions. Only a slight decrease in values towards weaker boundary conditions could be observed.

The solubility of fly ash shows a different trend. For BC1 and BC2, the amount of dissolved SiO_2_ is significantly higher than the amount of dissolved Al_2_O_3_. Only in BC3 could an opposite trend be observed, although the differences in dissolved oxides here are very small. BC4, where stirring was performed at room temperature, resulted in only 0.2% of the total soluble fraction. The Si/Al ratio calculated by these results leads to a range from 0.65 to 1.27 for metakaolin and 0.0 to 2.27 for fly ash, with higher ratios for more severe boundary conditions.

### 3.2. Workability of Fresh Paste

**Figure 3:**
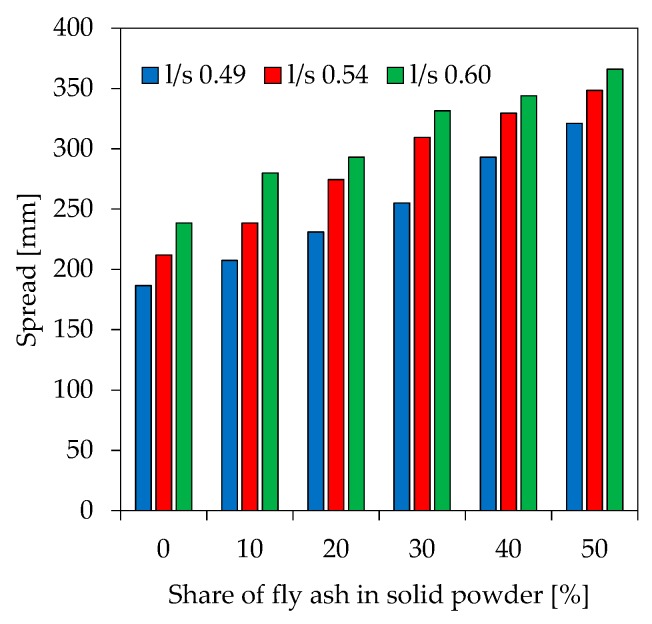
Spread of fresh geopolymer paste.

Workability of the pastes show a clear trend towards higher spreads with increasing l/s ratios and higher fly ash contents (Figure 3). Mixtures with l/s 0.49 have a spread in the range of 187 mm to 321 mm, 212 mm to 349 mm for l/s 0.54 and 239 mm to 366 mm for l/s 0.60, respectively. For the three different l/s ratios this results in almost linear correlations with coefficients of determination (R^2^) of 0.97 (l/s 0.49), 0.99 (l/s 0.54) and 0.99 (l/s 0.60).

### 3.3. Air Void Content of Fresh Paste

**Figure 4:**
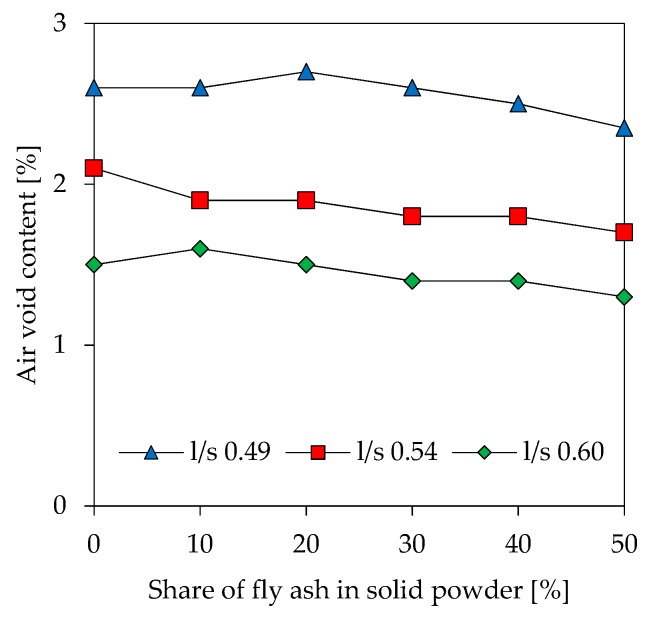
Air void content of fresh geopolymer paste.

Air void content of fresh pastes shows lower values when l/s increases (Figure 4). For l/s 0.49 the air void content is between 2.4% and 2.7%, resp. 1.7% to 2.1% for l/s 0.54 and 1.3% to 1.6% for l/s 0.60. For geopolymer blends with the same l/s value no significant differences were found with differing fly ash contents.

### 3.4. Isothermal Calorimetry

**Figure 5:**
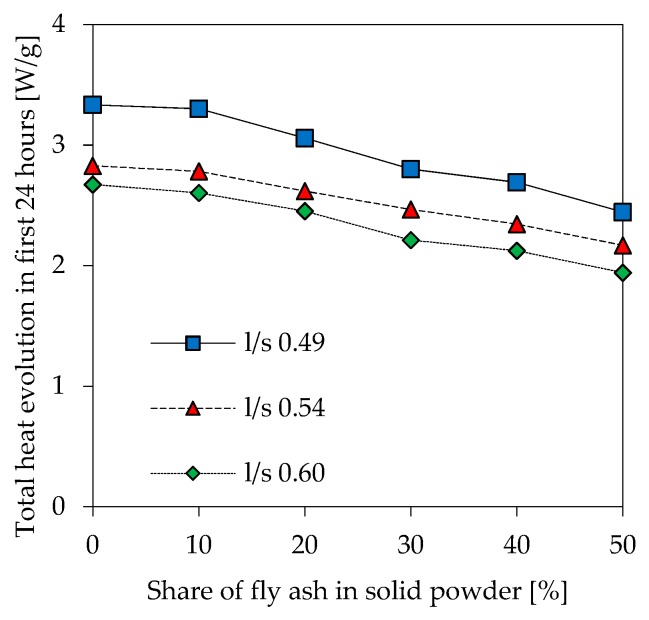
Total heat evolution of geopolymers in the first 24 h of reaction.

The heat evolution of all 18 geopolymer formulations reaches its maximum within the first 5 minutes of reaction. Total heat evolution within the first 24 h of reaction (Figure 5) shows a trend towards lower values for increasing l/s ratio and higher amounts of fly ash. The graphs for l/s 0.54 and l/s 0.60 are close to each other, whereas the lowest l/s ratio (0.49) results in a more pronounced difference of the total heat, especially for lower fly ash contents. 

### 3.5. Setting Time

Initial and final setting of geopolymers is presented in Table 3. For mixtures with l/s 0.49 initial setting of MK.49 starts after 92 minutes and increases steadily with fly ash addition until 232 minutes (FA50.49). 

Initial setting of l/s 0.54 mixtures starts at 127 minutes (MK.54) to 270 minutes (FA50.54), and 160 minutes (MK.60) to 327 minutes (FA50.60), respectively. Apart from the clearly differing initial setting times, there is only a slight deviation in time from initial to final setting, which is between 15 minutes and 43 minutes for l/s 0.49, 15 minutes and 30 minutes for l/s 0.54 and 23 and 40 minutes for l/s 0.60. The following linear correlations were calculated for the different setting times and fly ash contents:

(1) $$l/s\;0.49:\;s_{i}\left(\mathrm{fa}\right)=2.7\times \frac{\left(\mathrm{fa}\right)}{100}+86.0\;[\min]\;(R^{2}=0.97)$$

(2) $$l/s\;0.54:\;s_{i}\left(\mathrm{fa}\right)=2.7\times \frac{\left(\mathrm{fa}\right)}{100}+132.4\;[\min]\;(R^{2}=0.99)$$

(3) $$l/s\;0.60:\;s_{i}\left(\mathrm{fa}\right)=3.2\times \frac{\left(\mathrm{fa}\right)}{100}+155.1\;[\min]\;(R^{2}=0.97)$$

### 3.6. Compressive Strength

**Figure 6:**
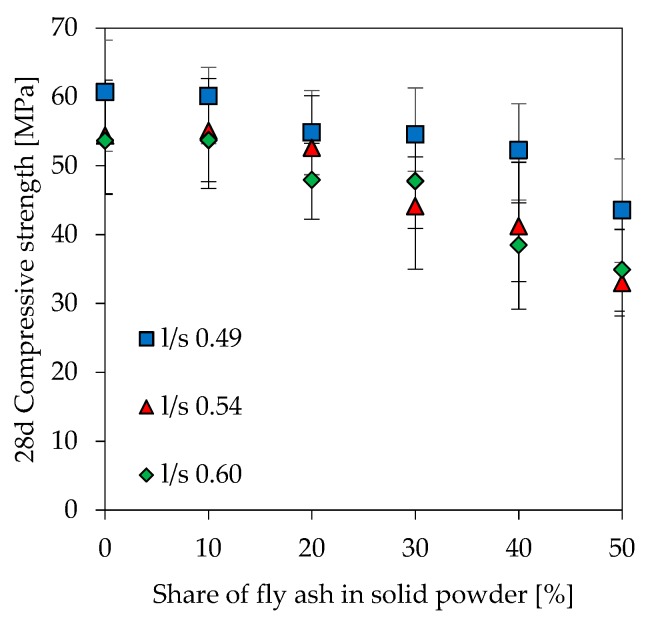
Compressive strength of geopolymer samples after 28 days of curing.

**Figure 7:**
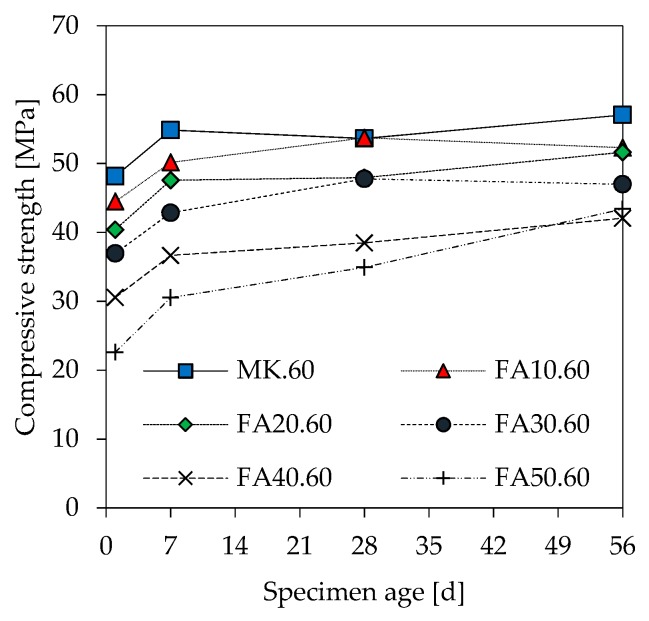
Evolution of compressive strength of geopolymer samples (l/s 0.60).

Figure 6 shows the compressive strength of geopolymers at 28 days. As expected, higher amounts of fly ash as well as an increase in l/s reduces compressive strength. For geopolymers with a constant l/s ratio, the compressive strength starts to decrease from a fly ash content of 20% onwards. Mixtures with 10% fly ash have almost the same strength as the corresponding metakaolin reference. Moreover, higher fly ash contents at very low l/s ratios (l/s 0.49) have a less pronounced effect on strength loss than is the case at higher l/s ratios. A similar trend on the strength loss of geopolymers after 1, 7 and 56 days (Figure 7) is observed for l/s 0.49 and l/s 0.54. Strength of geopolymers after 1 day of curing is in the range of 20 MPa to 50 MPa with lower strength at higher fly ash contents. Apart from FA50.60, most of the strength increase occurred within the first 7 days of curing. The percentage increase in strength from 1 day to 56 days for geopolymers is 18% (MK.60, FA10.60), 28% (FA20.60, FA30.60), 38% (FA40.60) and 92% (FA50.60). 

### 3.7. Porosity

**Figure 8:**
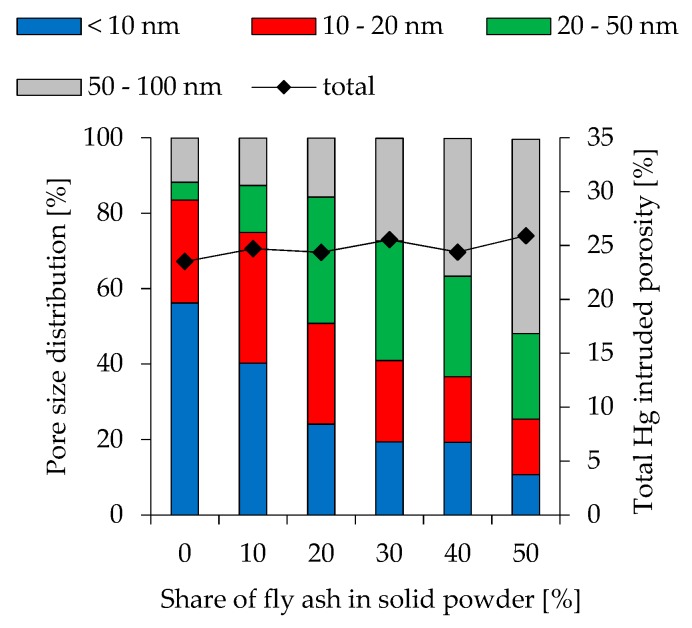
Pore size distribution of geopolymers cured for 28 days (l/s 0.49).

**Figure 9:**
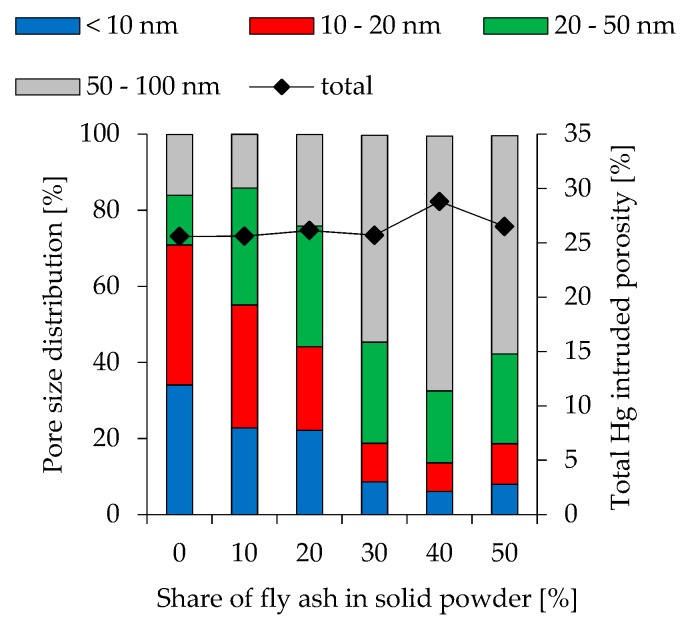
Pore size distribution of geopolymers cured for 28 days (l/s 0.54).

**Figure 10:**
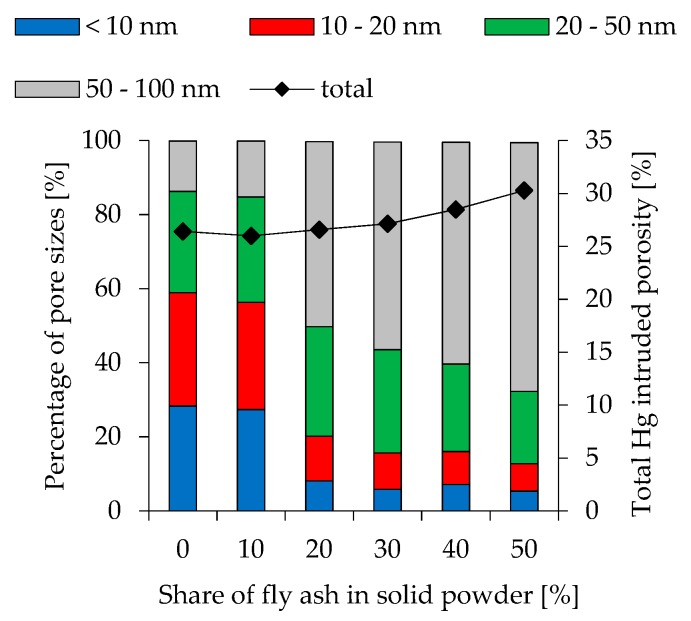
Pore size distribution of geopolymers cured for 28 days (l/s 0.60).

**Figure 11:**
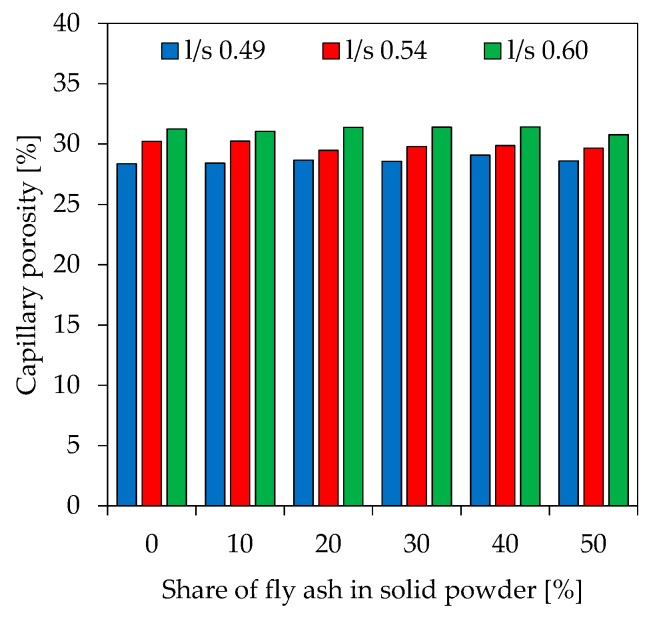
Capillary porosity of geopolymers (water adsorption tests).

To show the effect of changing pore size distribution at different fly ash contents and various l/s ratios, Hg intruded pores obtained by Mercury intrusion porosimetry (MIP) are divided into 4 different orders of magnitude (< 10 nm, 10 nm–20 nm, 20 nm–50 nm and 50 nm–100 nm). The results are presented in Figure 8 (l/s 0.49), Figure 9 (l/s 0.54) and Figure 10 (l/s 0.60), including also the total Hg intruded porosity. In addition to MIP, water adsorption tests were performed (see Figure 11) to evaluate the capillary porosity. 

Total Hg intruded porosity for l/s 0.49 mixtures is between 23.5% and 25.9% and increases at higher l/s ratios (25.6%–28.8% for l/s 0.54 and 26.0%–30.3% for l/s 0.60). Within these ranges higher fly ash contents lead to higher total porosity. Comparing various fly ash contents within a series of mixtures with constant l/s ratio, the amount of pores with a size of <10 nm reduces significantly at higher fly ash contents. This effect is most pronounced in geopolymers with l/s 0.49. Pores in the range of 10 nm–20 nm are also reduced but with a lower magnitude. The proportion of bigger pores increases accordingly. The influence of l/s ratio shows a similar trend, as higher ratios lead to an increase in bigger pores. Nevertheless, compared to the effect of fly ash, the effect of l/s is less pronounced. Geopolymers without fly ash show a strong effect of l/s on the pore size distribution only when MK.49 and MK.54 are compared, whereas the difference between MK.54 and MK.60 is less pronounced. Apart from FA20.54 and FA20.60, this can also be stated for the other fly ash contents, as the change in pore size distribution from l/s 0.54 to l/s 0.60 is obvious in almost all cases, whereas the difference of l/s 0.54 and l/s 0.60 is less pronounced. 

The capillary porosity resulting from water adsorption tests (Figure 9) reveal that the effect of increasing l/s ratios on total porosity is stronger than the one induced by higher fly ash contents. Capillary porosity for l/s 0.49 is between 28.4% and 29.1%, for l/s 0.54 between 29.5% and 30.2% and for l/s 0.60 between 30.8% and 31.4%, respectively. Table 4 shows the total porosity measured by MIP and the total capillary porosity measured from water absorption tests. Apart from FA50.60, the total capillary porosity is significantly higher than MIP porosity. 

### 3.8. Thermogravimetric Analysis-Differential Scanning Calorimeter (TGA-DSC)

**Figure 12:**
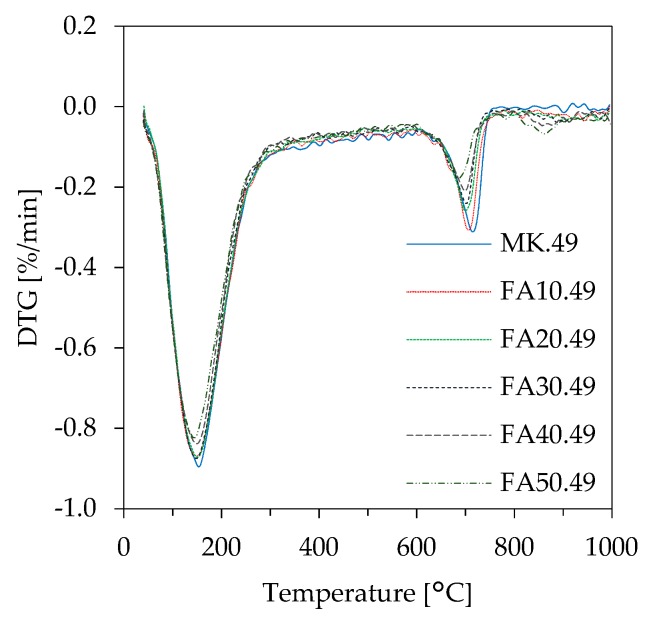
Differential thermogravimetric (DTG) curves of geopolymers (l/s 0.49).

**Figure 13:**
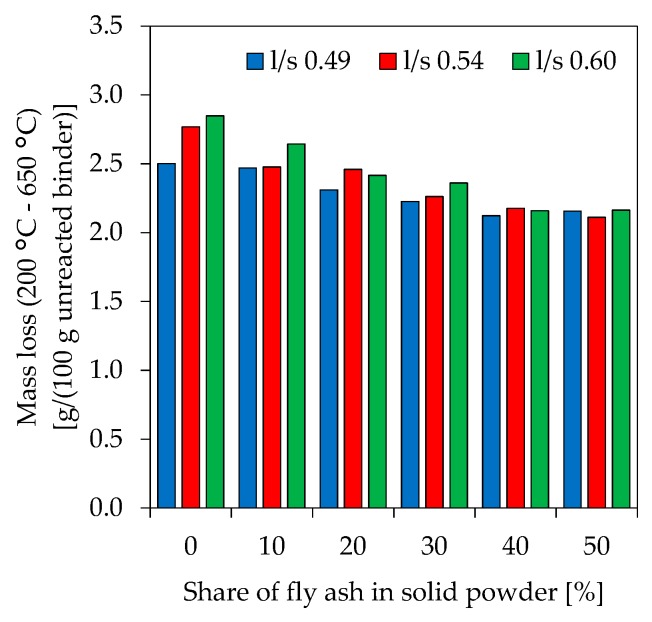
TGA (thermogravimetric analyzes) mass loss of geopolymers (temperature range 200 °C–650 °C).

In the present study, the evaluation of results obtained from TGA-DSC measurements (example of differential thermogravimetric (DTG) curves in Figure 12) is focusing on mass loss in the temperature range 200 °C to 650 °C (Figure 13), a range following the limits set by Douiri et al. [51] and Assaedi et al. [52]. The mass loss of geopolymers within the chosen ranges gives information about the degree of reactivity and the amount of actually newly build geopolymer gel. Figure 11 reveals that higher mass losses are associated with decreasing fly ash contents as well as increasing l/s ratios. The differences in mass loss are less pronounced for higher proportions of fly ash. 

### 3.9. Scanning Electron Microscopy with Energy Dispersive Spectroscopy (SEM-EDS)

**Figure 14:**
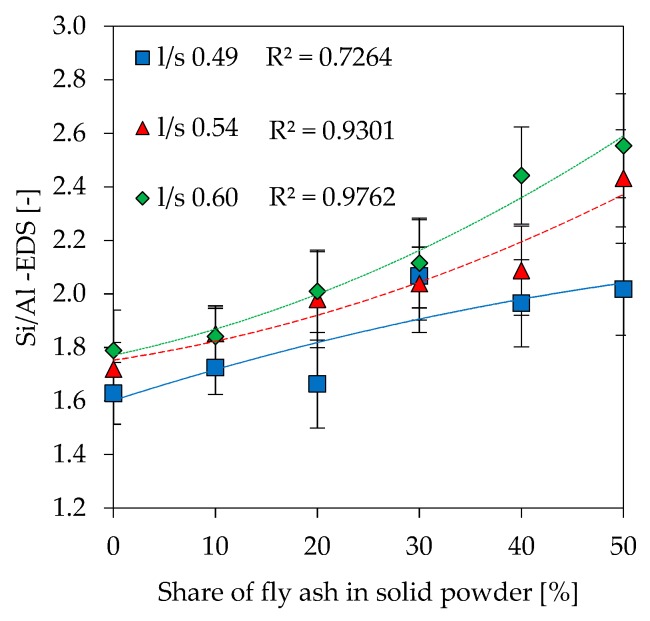
Si/Al ratio measured by energy dispersive spectroscopy (EDS) (Si/Al-EDS).

**Figure 15:**
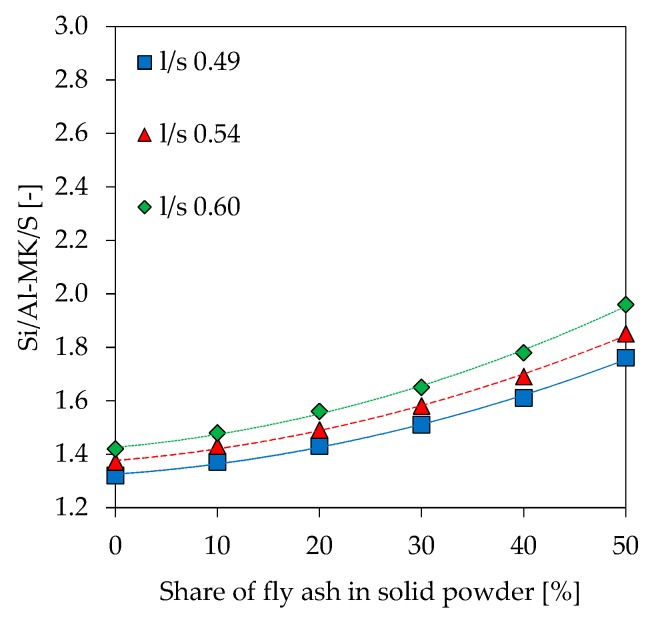
Theoretical Si/Al ratio, calculated with amorphous Si and Al, without fly ash (Si/Al-MK/S).

**Figure 16:**
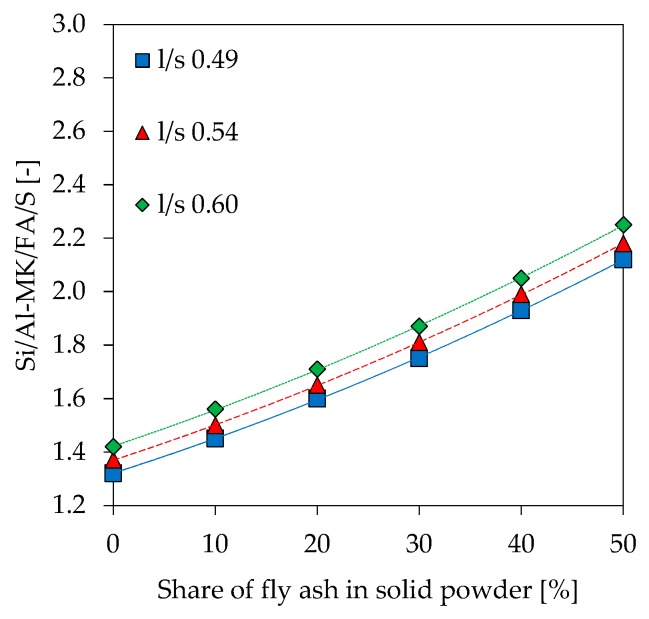
Theoretical Si/Al ratio, calculated with amorphous Si and Al, including fly ash (Si/Al-MK/FA/S).

**Figure 17:**
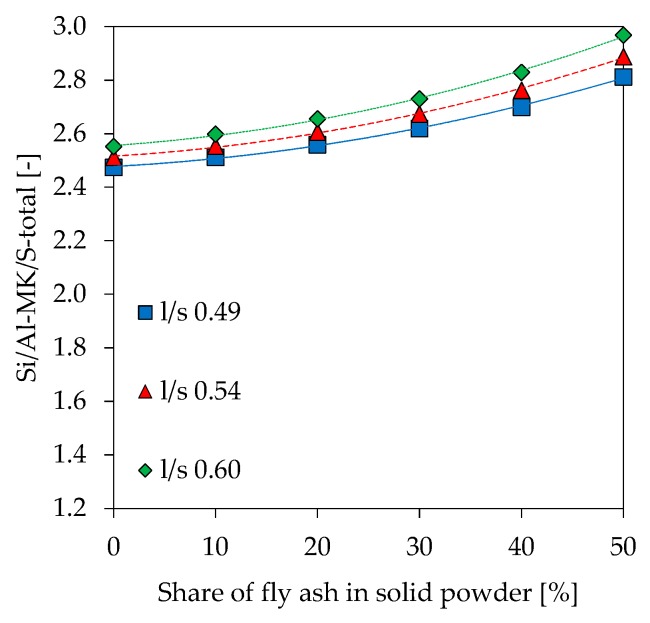
Theoretical Si/Al ratio, calculated with total amount of Si and Al, without fly ash (Si/Al-MK/S-total).

Si/Al ratios as results of SEM-EDS analyzes (Si/Al-EDS) are shown in Figure 14. In addition to the experimentally determined values, theoretical Si/Al ratios were calculated under following assumptions about amount of reactive Al and Si being incorporated in the gel:

1. Amorphous Si and Al portion from metakaolin and alkaline solution (Si/Al-MK/S) (Figure 15),
2. As in No. 1 plus amorphous Si and Al from fly ash (Si/Al-MK/FA/S) (Figure 16),
3. Total amount of Si and Al from metakaolin and alkaline solution (Si/Al-MK/S-total) (Figure 17).

The total amount of Si and Al results from the chemical composition of used materials, where the amorphous Si and Al is taken from Table 2. All graphs are presented including trendlines (polynomial functions of degree 2). Si/Al-EDS (Figure 12) shows an increase in the ratios for higher fly ash contents and higher amounts of potassium silicate solution in geopolymers. This is likely due to the lower amount of Al from metakaolin, as the mass percentage of the raw material decreases at higher substitution rates of fly ash and higher l/s ratios. In this context, Si/Al-EDS for l/s 0.49 shows a slightly different behavior, a convex instead of concave trendline, but exhibiting also a much lower coefficient of determination. Experimental Si/Al-EDS values are higher than Si/Al ratios calculated from the amorphous content of the raw materials with (Si/Al-MK/FA/S) and without fly ash (Si/Al-MK/S). The highest values for Si/Al-MK/S-total, higher than the measured and other calculated Si/Al ratios, results from the consideration of the total content of oxides. This, in the case of metakaolin, causes the Si value to rise sharply since the used metakaolin has a high content of crystalline quartz. In other words, measured values are in line with the theoretical cases and agree with results from reactivity of the precursors.

## 4. Discussion

Compared to pure metakaolin-based mixtures, blending fly ash to a metakaolin geopolymer leads to a loss of mechanical properties due to the low reactivity of fly ash and an increase in workability. Although the results from the dissolution tests cannot be directly linked to the reactivity potential of the powdery precursors within the geopolymer formulation, due to different alkaline solutions and concentrations of OH^-^, it shows a trend with regard to the significantly deviating initial (dissolution) reactivity of metakaolin and fly ash. This is also confirmed by the fact that fly ash geopolymers usually have to be post-treated at elevated temperatures, whereas metakaolin geopolymers harden under ambient conditions, if calcined properly. With regard to the dissolution tests, it can be seen that the solubility of metakaolin also depends on the boundary conditions of the tests, an effect that is lesser pronounced than in the case of fly ash (see Figure 1 and Figure 2). Especially in the context of Si/Al ratio calculated from the amount of dissolved Si and Al species, the deviation of Si/Al of metakaolin is less pronounced than the one of fly ash. Similar results have been published by Buchwald [53], confirming the significantly lower amount of dissolved Al from fly ash in comparison to the amount of dissolved Si. Temperature and concentration of the potassium hydroxide solution are key factors for activating resp. dissolving metakaolin and fly ash. At room temperature, significantly less oxides from metakaolin and fly ash are dissolved. In a geopolymer mixture, this can be compensated by using an alkaline silicate solution, as the dissolved Si in the activator represents an additional reaction partner which accelerates the polycondensation reaction [54]. The geopolymerisation reaction in the presence of a hydroxide solution is correspondingly slower and can extend over a significantly longer period of time [55,56]. Therefore, the results of the solubility tests correspond to the deviating reaction rates during alkaline activation with hydroxide solution and alkali silicate solution.

The influence of an increase of fly ash in geopolymer mixtures on setting and especially early compressive strength, therefore, will result in a lower overall reactivity of the material but also in a reduced amount of Al in the powder mixture. Higher amounts of reactive Al species usually lead to an increase in reactivity [57] as Al from powder precursors gets dissolved faster than Si [24]. In the process of geopolymerisation the polycondensation reaction between Al species and Si species progresses faster than the reaction between Si species [58]. In combination with an alkali silicate solution, the two reaction partners, which are required for an early reaction (Al and Si from metakaolin, Si from alkali silicate solution), are present. This leads to an increase in setting and faster strength development [57]. Similar results were published by de Silva et al. [4] and also explained by the more pronounced polycondensation reaction between Si and Al species compared to the reaction between Si species. Moreover, higher l/s ratios also lead to lower amount of reactive Al, in this context the total amount of water in the mixtures will however also play a crucial role [59].

Total heat evolved within the first 24 h of reaction (Figure 5) reaches its maximum when there is no fly ash in the mixture at all. This may be related to the lower amount of dissolved Al from metakaolin in blended geopolymer pastes, as discussed before. Lower amounts of evolved heat for geopolymers with higher amounts of fly ash and simultaneous reduction of the metakaolin content was also stated by Zhang et al. [41]. The decrease of total heat evolution at higher l/s ratios might also be due to the lower amount of Al species from powdery precursors. This explanation could be decisive for the initial reaction, as the Al species from the powdery precursor, compared to the Si species, are dissolved at a faster rate at the beginning of the reaction [24]. This might have a direct influence on the extent of the exothermic reaction of the dissolution process. However, total heat evolution comprises not only heat evolution from the dissolution of the powdery precursors but also from polymerization reaction [54,55,60]. Another possible explanation is the increase of the molar ratio K/Al (potassium/aluminium) at higher l/s ratios. K/Al ratios were calculated with amorphous (i.e., reactive) amount of Al in metakaolin (Table 2) and K from potassium silicate solution. For geopolymers MK.49, MK.54 and MK.60, which only comprise metakaolin as solid precursor, K/Al ratios are 1.16 (l/s 0.49), 1.29 (l/s 0.54) and 1.42 (l/s 0.60). Lower reactivity at higher Na/Al ratios, measured by isothermal calorimetry, was also reported by Zhang et al. [54,55]. Alkaline activation of metakaolin with Sodium hydroxide solution lead to a more pronounced reaction when Na/Al of the geopolymers was increased from 0.74 to 1.10, molar ratios higher than 1.10 resulted in lower heat evolution [55]. Although the type of activator and the alkali metal is different from the alkaline solution used in this study, this could provide an explanation for the decrease in heat evolution at higher l/s ratios. Zhang et al. [54,55] also conclude that the influence from Na/Al is more pronounced than the influence of Si/Al.

The effect of fly ash content and l/s ratio on compressive strength (see Figure 6) is very complex and requires the consideration of many contributing factors. The influence of reactive Al on early strength has already briefly been mentioned before. In this context, the Si/Al ratio of the geopolymer network has a decisive effect as higher ratios up to a certain degree usually lead to a more homogenous and denser matrix [4]. Bonds between Si species in the alumosilicate network are stronger than those between Si and Al [61] and therefore result in a higher strength of the network as well [5,6]. Literature reports values of 2.5 [62], 2.3 [63], 2.0 [64], 1.9 [5], [65], 1.7 [66], 1.7 to 1.9 [4] as well as 1.8 to 2.2 [67] as the optimal Si/Al ratios for metakaolin based geopolymers. The deviating values can be explained by the different specimen ages at the day of testing, as the geopolymer is initially characterized by an aluminum richer matrix and therefore lower Si/Al ratios [24]. Deviating solids contents in the formulations can also superimpose the influence of the Si/Al ratio [65]. 

In the present study, the negative influence of fly ash and higher l/s ratios on early and “final” strength after 56 days (see Figure 7) will probably superimpose the effect of Si/Al ratio. Si/Al ratios in this paper are measured by SEM-EDS and also calculated by taking into account different material compositions, which clearly show that both a higher fly ash content as well as increasing l/s ratios result in a lower strength, although the Si/Al ratios increase (see Figure 14). The more moderate but pronounced strength increase at higher fly ash contents is a known fact in cementitious systems, which is due to low reactivity of fly ash. In this context, however, higher strength with higher fly ash amounts can only be achieved when using reduced l/s ratios.

Higher amounts of larger pores for geopolymers with increasing fly ash content were also mentioned by Zhang et al. [41] as well as the increase off total porosity. However, the change in pore size distribution in the present study is much more pronounced (see Figure 8, Figure 9 and Figure 10), which may result from the high amount of quartz in metakaolin and its, therefore, lower water demand compared to the metakaolin used by the aforementioned researchers. Correlating total porosity and pore size distribution to the compressive strength, also for 28 day old specimens, is ambiguous, but may be assumed in some cases. Comparison of MK.60 and FA10.60 shows that total porosity and pore size distribution is almost identical (see Figure 10) as well as the compressive strength (see Figure 7). The same statement can be made for geopolymers FA20.60 and FA30.60. The obvious change in pore size distribution at the transition from 20% to 30% fly ash is also reflected in the results of the compressive strength. The influence of Si/Al ratio, therefore, could be little or superimposed by the influence of total porosity and pore size distribution, although Si/Al-EDS shows differences for both cases (MK.60 compared to FA10.60 and FA20.60 compared to FA30.60). Furthermore, it is noticeable that the Si/Al-EDS values at the transition from 20% to 30% fly ash also show a sizeable increase of the values, which was mentioned before for the pore size distribution.

Thermogravimetric analysis-differential scanning calorimeter (TGA-DSC) of geopolymer powder samples and the evaluation of the measurements in the range 200 °C–650 °C (see Figure 13) can be an indication of the amount of newly formed geopolymer gel. The results show that less water is bound at higher fly ash contents and increasing l/s ratios. In agreement with the reactivity of the fly ash as well as the overall amount of reactive phases, higher amounts of fly ash result in lower amounts of geopolymer gel and, therefore, less structural and/or chemically bound water. The effect of the l/s ratio can be explained by higher amounts of reactive Si from potassium silicate solution, which increase of the total amount of geopolymer gel. However, in the TGA-DSC measurements different processes may overlap. This complicates the allocation of the respective mass losses to certain processes chemically and physically conditioned as well as the temperature range at which those processes take place. Furthermore, the information given in literature differs greatly with regard to the cause of mass loss and the respective temperature range (Table 5). 

In addition to varying temperature ranges and their designations, the literature also mentions different maximum temperatures which, if exceeded, do not result in a further mass loss [51,69].

The calculated theoretical Si/Al ratios of geopolymers (see Figure 15, Figure 16 and Figure 17) as well as the measurements performed by SEM-EDS (see Figure 14) reveal challenges in the evaluation of this parameter. Each of the four Si/Al cases (measured SEM-EDS and the 3 calculation types) produce different results in terms of ratio values. In general, it is known that the Si/Al ratio of the geopolymer network differs from the one calculated by the (initial) composition of the geopolymer [70]. The higher values of Si/Al-EDS compared to the calculated values of the geopolymer mixtures can have several reasons. Firstly, it could be assumed that not all amorphous components of metakaolin are involved in the reaction [71]. Although the dissolution tests were not considered in the calculation of Si/Al, the results show that the dissolution behavior of the oxides may vary within the mixtures (see Figure 1 and Figure 2), since different boundary conditions also give different results. Furthermore, including results of the dissolution tests in the calculation of Si/Al is also a challenge, since the actual geopolymerisation reaction takes place at much lower l/s ratios compared to the conditions of the dissolution tests [72]. Considering fly ash in the calculation is also challenging as the oxides are in general not as easy to dissolve as the ones from metakaolin, nevertheless the higher strength increase for fly ash containing geopolymers (see Figure 7) indicate that the dissolution does take place to at least a certain extent. 

The participation of the crystalline quartz in the geopolymerisation process, which could theoretically influence the Si/Al ratio, could also not be determined within the scope of this paper. In this context, literature indicates that quartz is not substantially involved in the reaction [38,73,74], whereas Autef et al. [75,76] mention that the interaction of quartz with the geopolymer gel is at least possible. Other researchers who performed SEM-EDS measurements of the interfacial transition zone between geopolymer gel and aggregates state that higher Si/Al ratios or higher amounts of Si in this interface area result from the increased adsorption of Si from the alkaline silicate solution and not from the dissolution of the adjacent quartz particles [77]. 

## 5. Conclusion

Based on results in this paper, the following conclusions can be drawn:

1. Substituting an impure metakaolin by 10% of fly ash increases workability and retards initial and final setting without significantly affecting the strength and microstructure of the geopolymer, especially at higher l/s ratio.
2. Comparable pore size distribution (mercury intrusion porosimetry) between metakaolin geopolymer (0% fly ash content) and geopolymer with 10% fly ash are detected only at the highest l/s ratio (0.60). Lower l/s ratios show a significant differences between the two geopolymer formulations.
3. Above 20% fly ash content there are significant differences with regard to strength and porosity, whereby the change in pore size distribution (mercury intrusion porosimetry) is most pronounced.
4. Due to lower amounts of Al in geopolymers, total amount of heat evolved within the first 24 h of reaction decreases at higher l/s ratios and higher amounts of fly ash.
5. Si/Al ratios obtained by SEM-EDS are between the calculated extreme cases (full and partial/amorphous reactivity), and are in agreement with results from reactivity of the precursors tested in diluted KOH.

## Figures and Tables

**Table 1:** Amorphous amount (amor.), main crystalline phases and loss on ignition (LOI) of metakaolin (MK) and fly ash (FA).

	amor.	Quartz	Muscovite	Mullite	Calcite	Diaoyudaoite	Other (<0.5%)	LOI
MK	46.0	39.6	10.0	0.2	2.0	1.0	1.2	0.8
FA	72.0	9.5	0.0	16.0	0.0	0.0	2.5	4.5

**Table 2:** Amorphous Si, Al, SiO_2_, Al_2_O_3_ and molar Si/Al ratio of metakaolin (MK) and fly ash (FA), determined from powder X-ray diffraction (XRD) and X-ray fluorescence (XRF) results.

	Si [wt.%]	Al [wt.%]	SiO_2_ [wt.%]	Al_2_O_3_ [wt.%]	Si/Al (molar) [-]
MK	10.63	11.50	22.74	21.73	0.89
FA	18.44	6.74	39.46	12.74	2.63

**Table 3:** Initial and final setting of geopolymers.

		l/s 0.49				l/s 0.54				l/s 0.60			
	Fly ash [%]	0	10	30	50	0	10	30	50	0	10	30	50
Setting													
Initial		92	115	162	232	127	165	207	270	160	197	245	327
Final		125	157	177	267	147	180	237	295	190	220	270	362

**Table 4:** Comparison of total Hg intruded porosity (MIP) and capillary porosity (water adsorption).

		l/s 0.49				l/s 0.54				l/s 0.60			
	Fly ash [%]	0	10	30	50	0	10	30	50	0	10	30	50
Porosity													
Hg intruded [%]		23.5	24.7	25.5	25.9	25.6	25.6	25.7	26.5	26.4	26.0	27.1	30.3
Capillary [%]		28.4	28.4	28.6	28.6	30.2	30.2	29.8	29.6	31.3	31.0	31.4	30.8

**Table 5:** Temperature ranges and designation of the associated mass loss taken from literature.

Designation	Range [°C]	Reference
Physically adsorbed an interstitial evaporable water	0–120	Casarez et al. [68]
	0–150	Assaedi et al. [52]
	0–200	Douiri et al. [51]
Water from alumosilicate network	120–200	Casarez et al. [68]
Interstitial water	150–300	Assaedi et al. [52]
Water in nano pores	180–600	Škvára et al. [69]
Structural water	200–400	Douiri et al. [51]
Dihydroxylation of chemically bound water	300–600	Assaedi et al. [52]
Carbonation process	450–800	Casarez et al. [68]
Carbon remnants in fly ash	600–700	Assaedi et al. [52]
